# Mental stress induces endothelial dysfunction by AT1R-mediated redox imbalance in overweight/obese men

**DOI:** 10.1590/1414-431X2023e12547

**Published:** 2023-03-24

**Authors:** H.N.M. Rocha, G.M.S. Batista, A.S. Storch, V.P. Garcia, G.F. Teixeira, J. Mentzinger, E.A.C. Gomes, M.O. Campos, A.C.L. Nóbrega, N.G. Rocha

**Affiliations:** 1 Laboratório de Ciências do Exercício, Departamento de Fisiologia e Farmacologia, Universidade Federal Fluminense, Niterói, RJ, Brasil; 2 Instituto Nacional de Ciência e Tecnologia - (In)Atividade Física e Exercício, Conselho Nacional de Desenvolvimento Científico e Tecnológico, Universidade Federal Fluminense, Niterói, RJ, Brasil

**Keywords:** Mental stress, Endothelial function, Oxidative stress, Angiotensin II, Ascorbic acid, Obesity

## Abstract

The main goal of this study was to determine whether oxidative imbalance mediated by AT1 receptor (AT1R) is responsible for deleterious endothelial responses to mental stress (MS) in overweight/obese class I men. Fifteen overweight/obese men (27±7 years old; 29.8±2.6 kg/m^2^) participated in three randomized experimental sessions with oral administration of the AT1R blocker olmesartan (40 mg; AT1R blockade) or ascorbic acid (AA; 3g) infusion or placebo [both intravenously (0.9% NaCl) and orally]. After two hours, endothelial function was determined by flow-mediated dilation (FMD) before (baseline), 30 min (30MS), and 60 min (60MS) after a five-minute acute MS session (Stroop Color Word Test). Blood was collected before (baseline), during MS, and 60 min after MS for redox homeostasis profiling: lipid peroxidation (TBARS; thiobarbituric acid reactive species), protein carbonylation, and catalase activity by colorimetry and superoxide dismutase (SOD) activity by an ELISA kit. At the placebo session, FMD significantly decreased 30MS (P=0.05). When compared to baseline, TBARS (P<0.02), protein carbonylation (P<0.01), catalase (P<0.01), and SOD (P<0.01) increased during the placebo session. During AT1R blockade, FMD increased 30 min after MS (P=0.01 *vs* baseline; P<0.01 *vs* placebo), while AA infusion increased FMD only 60 min after MS. No differences were observed during MS with the AT1R blockade and AA regarding TBARS, protein carbonylation, catalase, and SOD. AT1R-mediated redox imbalances played an important role in endothelial dysfunction to mental stress.

## Introduction

Overweight/obesity is considered a major risk factor for noncommunicable diseases such as cardiovascular diseases, diabetes, and some types of cancer, contributing substantially to worldwide mortality (1). According to the World Health Organization, 39% of adults aged 18 years or over are overweight, while 13% have been diagnosed with obesity (2). In addition, psychological or mental stress (MS) is also an important risk factor for the development and progression of cardiovascular diseases (3), increasing the risk of acute coronary syndrome by 30% and the risk of stroke by 24% in men (4). Acute MS seems to lead to a transitory endothelial dysfunction both in health and disease (5,6) together with an impaired endothelial repair mechanism (7). Chronically, MS effects can be permanent for endothelial function, especially in individuals that already present risk factors such as overweight/obesity (8).

The mechanism by which MS leads to endothelial dysfunction in humans is still unknown. The renin-angiotensin system (RAS) activated by stress-mediated sympathoexcitation plays a central role in endothelial homeostasis (9). Angiotensin II (Ang II) is the main active mediator of the RAS classic pathway, which acts on endothelial and smooth muscle cells, through the angiotensin type 1 (AT1R) and type 2 receptors (10). *In vivo* and *in vitro* experimental studies have demonstrated that increases in Ang II - AT1R signaling leads to an imbalance of vasoactive substances, downregulating endothelial nitric oxide synthase, an enzyme that synthesizes nitric oxide (NO), and vasoconstrictor tone predominates (11). Also, Ang II - AT1R pathway seems to activate NADPH oxidase, increasing reactive oxygen species (ROS) and inflammation in overweight/obese adults (9,12). However, it is not clear whether AT1R-mediated oxidative stress is the underlying mechanism of endothelial responses to MS.

It has been demonstrated that both adults and children with obesity present diminished flow-mediated dilation (FMD), a proxy of endothelial function, at resting conditions (13-16). Sales et al. (6) highlighted that MS evokes acute transient reductions in FMD in obese adults with metabolic syndrome. It is worth noting that chronic stress in obese adults seems to double the cardiovascular morbidity and mortality compared to healthy individuals (17,18). Thus, it is critical to elucidate the impact of the Ang II-AT1R pathway on deleterious endothelial responses to MS in adults at increased cardiovascular risk, such as those with overweight/obesity.

Considering that Ang II modulates MS responses, it is believed that imbalances in redox homeostasis mediated by AT1R may be the underlying mechanism related to impaired stress-induced endothelial dysfunction in overweight/obese adults. Also, we hypothesized that AT1R blockade and ascorbic acid - free radical scavenger (antioxidant) - would similarly restore endothelial function in response to MS.

## Material and Methods

### Study population and protocol

Fifteen non-hypertensive overweight/obesity grade I men (27±7 years) were recruited from the local community. All individuals presented a BMI between 25 and 35 kg/m^2^ and body fat mass higher than 25%. Inclusion criteria included absence of any diagnosed disease, non-smoker status, and sedentary lifestyle (<150 min per week of moderate intensity cardiorespiratory exercise training) (19). All data collection took place at the Laboratory of Exercise Sciences (Niteroi, Brazil) during August 2017 and October 2018. This study protocol was approved by the Ethics Committee of Fluminense Federal University (CAAE 76594217.0.0000.5243) and by the Brazilian Clinical Trials Registry (Rio de Janeiro, RJ; REQ: 9237; www.ensaiosclinicos.gov.br) and conformed to the standards set by the latest revision of the Declaration of Helsinki. All subjects gave written informed consent before their participation in the study.

Biochemical analyses were conducted at the first visit. The subjects were then invited to a second visit that consisted of eligibility screening, i.e., clinical history assessment, anthropometric and arterial pressure measurements, resting electrocardiogram, and biochemical blood analysis interpretation. When the inclusion criteria were met, subjects were invited to three experimental sessions, with at least seven days between them.

The protocol consisted of a randomized, 3-way crossover, blind, placebo-controlled study. Experimental sessions consisted of oral administration of angiotensin II type 1 receptor blocker (AT1R blockade; 40 mg, olmesartan (OLM), lot number: 60818, Pfizer, USA), ascorbic acid (AA; 3 g diluted in 500 mL of 0.9% NaCl) administered intravenously for 30 min, or placebo [both intravenously (0.9% NaCl) and orally]. In the AT1R blockade session, an olmesartan pill was offered and saline infusion was performed; in the AA session, a placebo pill was offered and AA infusion was performed; in the placebo session, a placebo pill was offered and saline infusion was performed. All subjects were instructed to avoid alcohol, caffeine, and intense exercise in the 48 h prior to the visits. In addition, subjects were advised to follow a low-nitrate, low-nitrite diet prescribed by a nutritionist in the 24 h prior to the sessions. Mainly, subjects were advised to avoid red meat, fish, dark green vegetables, citrus fruits, oilseeds, and highly processed food.

**Figure 1:**
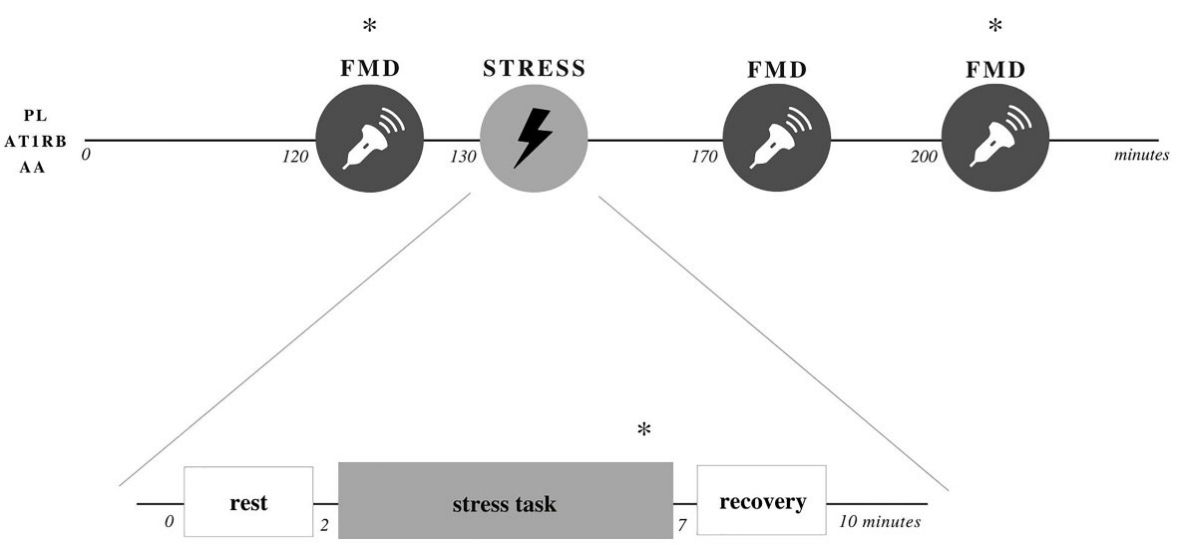
Experimental protocol. Asterisks indicate blood sampling. FMD: flow-mediated dilation; PL: placebo; AT1RB: angiotensin II type 1 receptor blockade; AA: ascorbic acid.

The experimental sessions took place in the morning in a climate-controlled environment (22-24°C). After the subjects arrived, blood pressure was measured in the seated position, and the subject was instructed to lie on the stretcher for drug administration. At this time, an intravenous catheter was placed in the antecubital cavity for blood sampling to evaluate endothelial biomarkers and oxidative stress, and a blind oral administration of AT1R blocker or an intravenous administration of AA or placebo was performed. All participants were submitted to the three conditions, with an interval of at least seven days between them. Subjects then rested supine for two hours, the time required for the blocker to reach the peak of action (20). Following the resting period, brachial artery FMD was assessed in the dominant arm (baseline). Subsequently, subjects were submitted to a 5-min MS task. FMD was conducted again 30 (30MS) and 60 min (60MS) after MS. Venous blood samples were also collected before (baseline), during (MS), and 60 min (60MS) after MS (Figure 1). Immediately after sampling, each blood tube was centrifuged according to the specific requirements of each variable, and the plasma was aliquoted and snap-frozen. At the time of analyses, the aliquots were thawed at room temperature and discarded after use.

### Biochemical analyses

Blood samples were drawn from the antecubital vein after 12-h fasting for the following measurements: fasting glucose, total cholesterol, high-density lipoprotein (HDL)-cholesterol, triglycerides, and insulin using enzymatic colorimetric methods. Very low-density lipoprotein (VLDL)-cholesterol values were calculated based on triglyceride values, and low-density lipoprotein (LDL)-cholesterol was calculated by the Friedewald equation, which is based on total cholesterol, HDL-cholesterol, and triglyceride values.

### Bioimpedance

Body composition by bioelectrical impedance analysis predicts the percentage of lean mass, fat mass, and total water volume (extracellular and intracellular) through an electrical current generated and detected by electrodes. Two electrodes each were positioned in the metacarpal and metatarsal, discharging an electric current of 50 kHz generated by an external source (Quantum II - Body Composition Analyzer; RJL Systems, USA) (21). This current was detected by two other electrodes positioned in the wrist and ankle, evaluating the change in initial frequency. The impedance and reactance data provided by the source were analyzed using the RJL Systems Body Composition software.

### Mental stress

The MS task applied was an adapted version of the Stroop Color Word Test (22), which consists of a slideshow projected on the ceiling above the subject that changes every two seconds. In addition, auditory conflicts were continuously inflicted via headphones using a standardized audio clip of three different people (two men and one woman) saying names of colors. The colors mentioned in the audio were in a different order then those presented in the slideshow. MS tasks consisted of two minutes of baseline measurements, five minutes of MS, and three minutes of recovery after the test, during which the subject rested quietly. Non-invasive beat-by-beat blood pressure and heart rate were recorded via photoplethysmography on the middle finger (Finometer, Finapres Medical Systems, The Netherlands). The level of perceived stress was assessed after each test using a subjective scale from zero to four, as follows: 0=non-stressful, 1=not very stressful, 2=stressful, 3=very stressful, and 4=extremely stressful. Blood flow measurements were performed at baseline, in the last 30 s of the first three minutes of MS, and in the last minute of recovery. Blood sampling for evaluation of endothelial biomarkers and oxidative stress was performed in the last two minutes of the MS task (Figure 1).

### Flow-mediated dilation

Brachial artery FMD was measured on the dominant arm before and 30 and 60 min after the MS task. Of all fifteen subjects, FMD was performed on the left arm of only two. Subjects adopted the supine position with the shoulder abducted at 80°. The forearm position was determined and held to optimize brachial artery imaging. In accordance with the most recent FMD guidelines (21), a rapid inflation/deflation pneumatic cuff (E-20 Rapid Cuff Inflator, D.E. Hokanson, USA) of appropriate size was placed around the forearm immediately distal to the olecranon process. Brachial artery imaging was obtained on the distal third of the arm (2-12 cm above the antecubital fossa) using a multifrequency linear-array (8-12 MHz) probe coupled to a high-resolution Doppler ultrasound system (LogiQ P5, GE Medical Systems, USA). Diameter and blood velocity were simultaneously acquired in duplex mode at a pulsed frequency of 30 MHz and adjusted to the full vessel width (insonation angle ≤60°). Baseline diameter and mean blood velocity waveforms were continuously recorded for 30 s. The cuff was then rapidly inflated to 220 mmHg for five minutes. After this period, the cuff was rapidly deflated. Doppler recordings resumed 15 s before cuff deflation and continued for three minutes. Brachial artery diameter was analyzed offline with an automated edge-detection and wall-tracking software (Vascular Research Tools 5, Medical Imaging Applications, USA). Regions of interest were identified and kept for the remaining analyses (6,23).

### Oxidative stress

Oxidative stress was determined by the measurement of lipid peroxidation markers (thiobarbituric acid reactive species, TBARS), protein oxidation (protein carbonylation) concentrations, and the activity of catalase and superoxide dismutase (SOD) (antioxidant enzymes), in plasma isolated from venous blood samples collected in EDTA tubes, which were centrifuged at 1050 *g* for 15 min at 20°C.

#### Lipid peroxidation

The evaluation of lipid peroxidation was performed by determining the levels of TBARS. This method is based on the reaction between two molecules of thiobarbituric acid (TBA) and one of malondialdehyde (MDA) resulting from lipid peroxidation and producing a complex (MDA:TBA) of pink color. To this end, 100 μL of serum was homogenized with 50 μL of SDS (8.1%), 550 μL phosphoric acid (1%), and 300 μL of thiobarbituric acid (0.6%). This solution was then heated to 95°C for 1 h in a dry bath and then centrifuged (2000 *g*) for 5 min at 25°C. The supernatant was used to quantify the TBARS levels. Plasma concentrations of lipoperoxides are reported in terms of MDA (nmol/mL) and determined in duplicate by TBARS measurement using a fluorimetric method (CV: 10.58%). The absorbance of each test was obtained in a 96-well microplate reader (Synergy H1 Hybrid Multi-mode, Biotek; USA) at 532 nm. This method used the substance 1,1,3,3-tetramethoxypropane to make the standard curve (24).

#### Protein carbonylation

The quantification of protein carbonylation was accomplished through the reaction of 2,4-dinitrophenylhydrazine (DNPH) with the carbonyls of oxidized proteins. In this assay, the total protein concentration was determined in duplicate (CV: 22.02%) according to the method of Lowry et al. (25) using a standard curve of albumin. The carbonyl concentration values were normalized by mg of albumin and are reported as nmol/g.

#### Catalase activity

The catalase enzymatic activity was determined in duplicate (CV: 17.03%) by colorimetric assay (Catalase Assay kit, USA), using plasma isolated from venous blood samples collected in EDTA tubes, according to manufacturer's instructions.

#### Superoxide dismutase activity

SOD activity was determined by an enzyme-linked immunosorbent assay (ELISA) kit (Human SOD2/Mn-SOD DuoSet ELISA Kit, R&D Systems, USA), using plasma isolated from venous blood samples collected in EDTA tubes, according to manufacturer's instructions.

### Calculations and statistical analysis

After analyzing brachial artery diameter and blood velocity, blood flow was calculated from the mean blood velocity and vessel area, considering 60 as a constant (i.e., V_mean_ × Area × 60). Shear rate (SR), a proxy of shear stress, was calculated as four times the ratio between mean blood velocity (V_mean_) and the artery diameter [i.e., 4 × (V_mean_/diameter)]. The area under the curve (AUC) was obtained from the cumulative SR during FMD from post-occlusion until peak diameter. Vascular conductance was calculated from mean blood flow and mean arterial pressure (mL·min·mmHg^-1^). Because baseline diameter (D_base_) could bias the FMD%, which is a ratio between peak diameter (D_peak_) and D_base_, the allometric scale proposed by Atkinson and Batterham (26) was used to account for possible baseline interferences. The regression's slope between logarithmically transformed D_peak_ and D_base_ was calculated [placebo, β=0.97 (95%CI: 0.897 to 1.047); AT1R blockade, β=0.96 (95%CI: 0.879 to 1.048); AA, β=0.96]. A regression slope smaller than one suggests that D_peak_ and D_base_ do not increase proportionally, meaning that the assumptions made based on FMD% might be biased. Then, logD_base_, logD_peak_, and the difference between them (D_peak_-D_base_) (logD_diff_) were entered into a multivariate general linear model considering logD_diff_ as the dependent variable, session (placebo or AT1R blockade) as the fixed factor, and logD_base_ as the covariate. Adjusted means were then antilog-transformed, subtracted by a value of 1 and multiplied by 100 to facilitate interpretation as percentage.

Considering the FMD results as the main outcome and the alpha error of 0.05, the power of the statistical test for a sample size of 14 individuals was 0.8. The Shapiro-Wilk test and homoscedasticity were performed by the Levene's test to verify the normal distribution of the variables. Two-way ANOVA was then performed for repeated measurements, where “condition” and “moment” were considered as the main factors. When significant differences were found for group, time, and/or interaction, Fisher's test was used as a *post hoc* procedure. The paired Student's *t*-test was carried out to compare the magnitude of response to MS between both sessions. Data are reported as means±SD. A probability less than or equal to 5% was considered statistically significant in two-tailed analyses. The statistical package used was Statistica (version 10.0, StatSoft Inc. 2011, USA).

## Results

The anthropometric, clinical, and biochemical profiles are presented in Table 1. As expected, all subjects presented a BMI between 26.7 and 34 kg/m^2^, characterizing the overweight/obesity grade I criteria, and a body fat mass higher than 27%.

**Table 1:** Anthropometric, hemodynamic, and biochemical profile of participants.

Variables		Reference values
Overweight (n)	8	-
Obese (n)	7	-
Age (years)	27±7	-
Weight (kg)	91.7±10.2	-
Height (cm)	175±0.08	-
BMI (kg/m^2^)	29.8±2.51	25-34.9
Body fat (%)	31.7±3.62	12-20
Waist circumference (cm)	99.0±5.9	90-110
SBP (mmHg)	123±7	≤120
DBP (mmHg)	80±8	≤80
Heart rate (bpm)	72±11	60-100
Glucose (mg/dL)	87.4±7.76	65-99
Insulin (uIU/mL)	12.9±6.19	1.9-23
HOMA-IR	2.98±1.42	<4.5
HOMA-β	192.70±105.08	167.0-175.0
Total cholesterol (mg/dL)	185.45±41.17	<190
HDL (mg/dL)	41.18±7.54	>40
LDL (mg/dL)	120.54±35.32	<130
VLDL (mg/dL)	23.83±9.74	2-30
TG (mg/dL)	109.36±57.1	<150

Data are reported as means±SD for a total of 15 participants. BMI: body mass index; SBP: systolic blood pressure; DBP: diastolic blood pressure; HDL: high-density lipoprotein cholesterol; LDL: low-density lipoprotein cholesterol; VLDL: very-low-density lipoprotein cholesterol; TG: triglycerides.

Regarding MS responses, according to the subjective scale used, the average level of perceived stress in all sessions was 2 (stressful). Table 2 shows systolic blood pressure (SBP), diastolic blood pressure (DBP), mean blood pressure (MBP), heart rate (HR), blood flow, and vascular conductance at baseline, during MS, and during recovery. There was a significant increase in the hemodynamic variables SBP, DBP, MBP, and HR (P<0.05 *vs* baseline) during MS in all sessions. In recovery, these variables decreased to baseline levels (P<0.01 *vs* MS). No differences were observed regarding blood flow and conductance. It is noteworthy that no differences were observed in the magnitude of response of hemodynamic variables between sessions, indicating that MS caused the same effect in all experimental sessions. Also, no differences were observed in regards to the direct effect of medication on hemodynamic variables, as we can attest by the lack of difference between the baseline moments of each session (Table 2). Moreover, none of the subjects enrolled in the present study reported adverse effects during any of the sessions.

**Table 2:** Hemodynamic parameters at baseline, during mental stress, and during recovery in overweight/obesity individuals after drug intervention.

Variables	Baseline	Mental Stress	Recovery
Placebo			
SBP (mmHg)	123±9	136±10*	124±10
DBP (mmHg)	76±8	88±10*	77±7
MBP (mmHg)	91±8	104±10*	93±8
Heart rate (bpm)	62±8	75±10*	64±8
Blood flow (mL/min)	179.89±77.49	224.37±102.31	232.63±141.96
Conductance (mL·min^-1^·mmHg^-1^)	1.96±0.77	2.15±0.94	2.53±1.42
AT1R blockade			
SBP (mmHg)	120±8	133±10*	123±8
DBP (mmHg)	75±9	88±8*	75±7
MBP (mmHg)	87±17	99±19*	92±6
Heart rate (bpm)	63±8	76±10*	65±9
Blood flow (mL/min)	193.78±134.41	257.68±129.60	203.05±177.91
Conductance (mL·min^-1^·mmHg^-1^)	2.07±1.38	2.47±1.24	2.35±1.86
Ascorbic acid			
SBP (mmHg)	124±9	140±6*	126±10
DBP (mmHg)	78±8	91±6*	80±8
MBP (mmHg)	94±8	107±6*	95±8
Heart rate (bpm)	65±11	76±11*	65±8
Blood flow (mL/min)	204.63±159.67	306.33±161.24	253.44±257.29
Conductance (mL·min^-1^·mmHg^-1^)	2.22±1.38	2.95±1.41	2.73±2.52

Data are reported as means±SD. SBP: systolic blood pressure; DBP: diastolic blood pressure; MBP: mean blood pressure; AT1R: angiotensin II type 1 receptor. *P<0.05 *vs* baseline and recovery within session (ANOVA).

**Figure 2:**
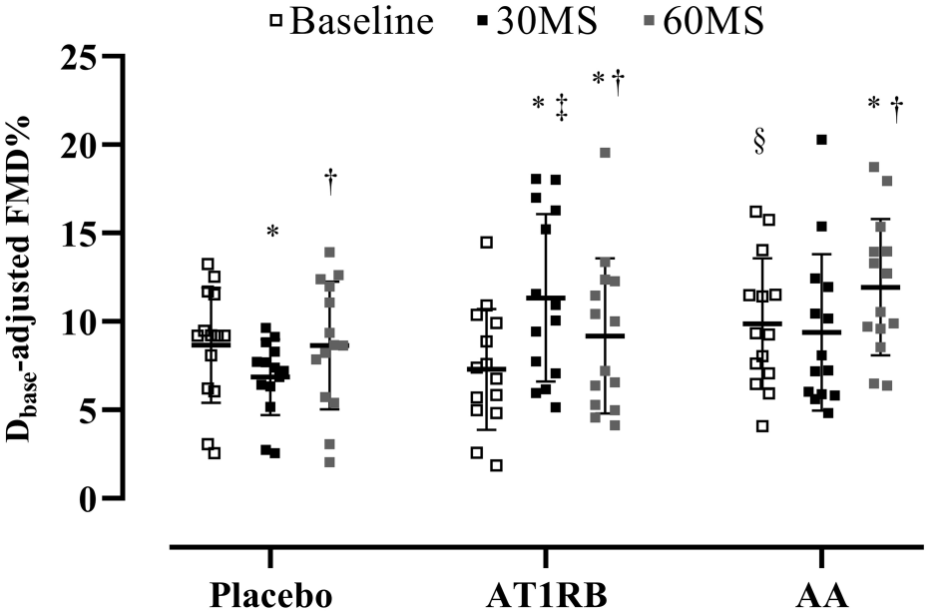
Flow-mediated dilation (FMD) before (baseline), 30 (30MS), and 60 min (60MS) after mental stress in overweight/obese individuals after oral administration of placebo, AT1R blockade, or AA. Vertical lines indicate means and SD. *P<0.05 *vs* baseline; ^†^P<0.05 *vs* 30MS; ^‡^P<0.05 *vs* placebo at the same moment; ^§^P<0.05 *vs* AT1R at the same moment (ANOVA). AT1RB: angiotensin II type 1 receptor blockade; AA: ascorbic acid.

Regarding endothelial function, D_base_-adjusted FMD decreased 30 min after MS (baseline, 8.73±1.03% *vs* 30MS, 7.49±1.03%; P=0.05) during the placebo session but increased 60 min after the stress task (30MS, 7.49±1.03% *vs* 60MS, 9.93±1.03%; P<0.02). During the AT1R blockade session, FMD increased significantly in response to MS (baseline, 7.60±1.02% *vs* 30MS, 10.66±1.03%; P<0.01), and it was different from placebo (30MS, 10.66±1.03%; P<0.01). Baseline FMD was decreased compared to AA (OLM, 7.60±1.02% *vs* AA, 9.33±1.03%; P<0.02). Also, FMD decreased 60 min after MS (30MS, 10.66±1.03% *vs* 60MS, 9.89±1.03%; P=0.03), but it was still higher compared to baseline (P<0.01). As for the AA session, D_base_-adjusted FMD at 60MS was improved compared to baseline (baseline, 9.25±1.02% *vs* 60MS, 11.03±1.03%; P<0.04) and 30MS (30MS, 9.41±1.02%; P<0.01) (Figure 2).

Table 3 provides the results of resting diameter (cm), peak diameter (cm), FMD%, AUC_SR_, and FMD%/AUC_SR_. The resting diameter was smaller at the placebo session compared to AT1R blockade, leading to a baseline difference in resting diameter (P<0.01), whilst baseline resting diameter at the AA session was smaller than baseline in the AT1R blockade. There was a baseline difference between FMD% during placebo and AT1R sessions (P<0.04). As expected, peak diameter was significantly higher than resting diameter at all times during the three sessions (P<0.01). At the placebo session, peak diameter at 30MS was lower compared to baseline (P<0.04) and 60MS (P<0.02), which was higher than baseline (P<0.02). No differences regarding FMD% were observed in the placebo session. While AUC_SR_ did not change during the placebo session, FMD%/AUC_SR_ was significantly decreased at 30MS compared to baseline (P<0.02).

**Table 3:** Flow-mediated dilation at baseline and 30 and 60 min after mental stress (MS) in overweight/obesity individuals after oral administration of placebo and AT1R blocker and infusion of ascorbic acid.

Variables	Baseline	30MS	60MS
Placebo			
Resting diameter (cm)	0.407±0.57	0.408±0.56	0.409±0.53
Peak diameter (cm)	0.441±0.55†	0.436±0.55* †	0.446±0.55* † §
FMD (%)	9.06±2.79	7.22±1.94	9.45±3.85
AUC_SR_ (10^-3^·s^1^)	20.79±10.36	24.78±15.02	20.98±9.11
FMD%/AUC_SR_ (%·10^-3^·s^1^)	5.17±4.01	3.46±1.98*	4.14±2.00
AT1R blockade			
Resting diameter (cm)	0.416±0.64^#^	0.403±0.62*	0.408±0.59*
Peak diameter (cm)	0.443±0.63†	0.446±0.57†	0.444±0.57†
FMD (%)	6.69±3.54^#^	11.20±5.03* ^#^	9.47±4.38*
AUC_SR_ (10^-3^·s^1^)	23.93±18.62	22.05±9.13	23.79±9.07
FMD%/AUC_SR_ (%·10^-3^·s^1^)	4.79±5.18	5.88±4.02^#^	4.07±2.25
Ascorbic acid			
Resting diameter (cm)	0.391±0.50‡	0.391±0.48	0.385±0.49* # §
Peak diameter (cm)	0.429±0.46†	0.427±0.56†	0.430±0.48† ^#^
FMD (%)	9.39±3.34	9.54±4.56# ‡	12.20±3.88* §
AUC_SR_ (10^-3^·s^1^)	20.20±10.70	18.56±7.47	22.47±9.77
FMD%/AUC_SR_ (%·10^-3^·s^1^)	5.55±3.55	5.19±2.550^#^	6.53±3.26# ‡

Data are reported as means±SD. FMD: flow-mediated dilation; AUC: area under the curve; SR: shear rate; 30MS: 30 min after mental stress; 60MS: 60 min after mental stress; AT1R: angiotensin II type 1 receptor. *P<0.05 *vs* baseline within session; †P<0.05 vs resting diameter; ^#^P<0.05 *vs* placebo at the same moment; ^‡^P<0.05 vs AT1R blockade at the same moment; ^§^P<0.05 *vs* 30MS within session (ANOVA).

Regarding the AT1R blockade session, resting diameter presented the same behavior as peak diameter during the placebo session, although peak diameter did not change during AT1R blockade. In relation to FMD%, endothelial function was improved at 30 min compared to baseline (P<0.01) and to the same moment in the placebo session (P<0.02). At 60 min, FMD% was still increased compared to baseline (P<0.02). AUC_SR_ did not change during the AT1R blockade session. However, FMD%/AUC_SR_ was increased at 30MS compared to placebo (P<0.02).

During the AA session, baseline resting diameter was lower compared to the same moment in the AT1R blockade session (P<0.01). At 60 min, resting diameter was lower than baseline (P<0.01) and at 30 min during the same session (P<0.02), and lower than the same moment in the placebo session (P<0.01). FMD% at 30 min was higher compared to the same moment in the placebo session (P<0.04) but was lower compared to the AT1R blockade session (P<0.02). At 60 min, FMD% was improved compared to baseline (P<0.02) and 30 min (P<0.03) during AA infusion. Similar to the AT1R blockade session, AUC_SR_ did not change during the AA blockade session, but FMD%/AUC_SR_ was increased at 30 min compared to placebo (P<0.02). Moreover, at 60 min, FMD%/AUC_SR_ was significantly greater in the AA session than in the placebo and AT1R blockade sessions.

**Figure 3:**
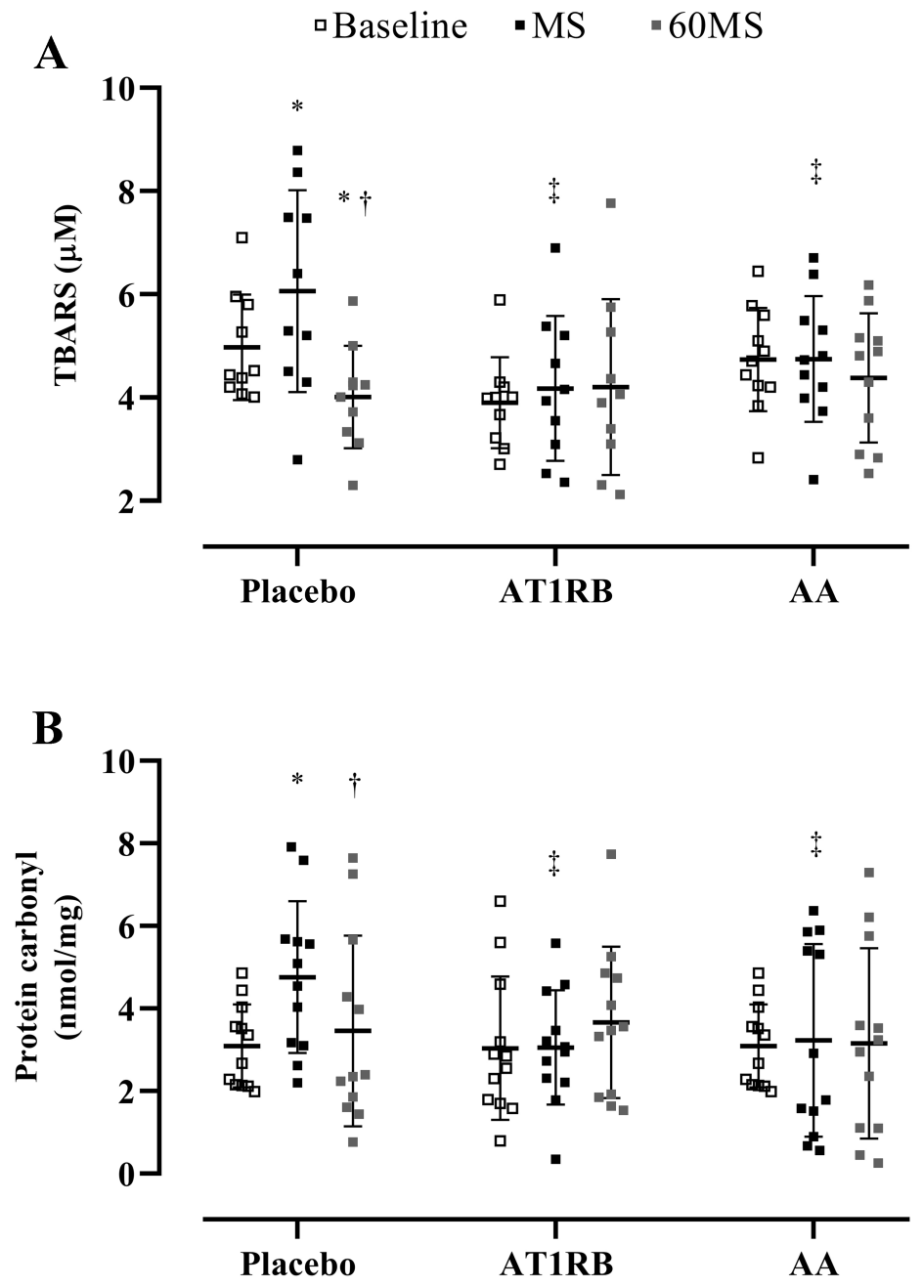
Lipid peroxidation (**A**) and carbonylated proteins (**B**) before (baseline), during (MS), and 60 min (60MS) after mental stress in overweight/obesity individuals after oral administration of placebo, AT1R blockade, and AA. Vertical lines indicate means and SD. *P<0.05 *vs* baseline; ^†^P<0.05 *vs* 60MS; ^‡^P<0.05 *vs* placebo at the same moment. FMD: flow-mediated dilation; AT1RB: angiotensin II type 1 receptor blockade; AA: ascorbic acid.

In the placebo session, lipid peroxidation was increased in response to MS (baseline, 4.97±1.02 nmol/mL *vs* MS, 6.06±1.95 nmol/mL, P<0.02; placebo, 6.06±1.95 nmol/mL *vs* AT1R blockade, 1.08±0.43 nmol/mL, P<0.01; placebo, 6.06±1.95 nmol/mL *vs* AA, 4.74±1.21 nmol/mL, P<0.01) but greatly decreased 60 min after (baseline, 4.97±1.02 nmol/mL *vs* 60MS, 4.01±0.99 nmol/mL, P<0.03; MS, 6.06±1.95 nmol/mL *vs* 60MS, 4.01±0.99 nmol/mL, P<0.01). During the AT1R blockade session and AA sessions, no differences were observed, meaning that both prevented lipid peroxidation increase during MS (Figure 3A). Protein carbonylation was also increased during MS (baseline, 2.95±1.09 nmol/g *vs* MS, 4.52±1.95 nmol/g; P<0.01) and returned to baseline levels 60 after MS during the placebo session (MS, 4.52±1.95 nmol/g *vs* 60MS, 3.16±2.45 nmol/g; P<0.03). Similar to lipid peroxidation, AT1R blockade and AA prevented protein carbonylation increase during MS (placebo, 4.52±1.95 nmol/g *vs* AT1R blockade, 3.05±1.38 nmol/mL, P<0.01; placebo, 4.52±1.95 nmol/g *vs* AA, 3.22±2.33 nmol/mL, P<0.01) (Figure 3B).

**Figure 4:**
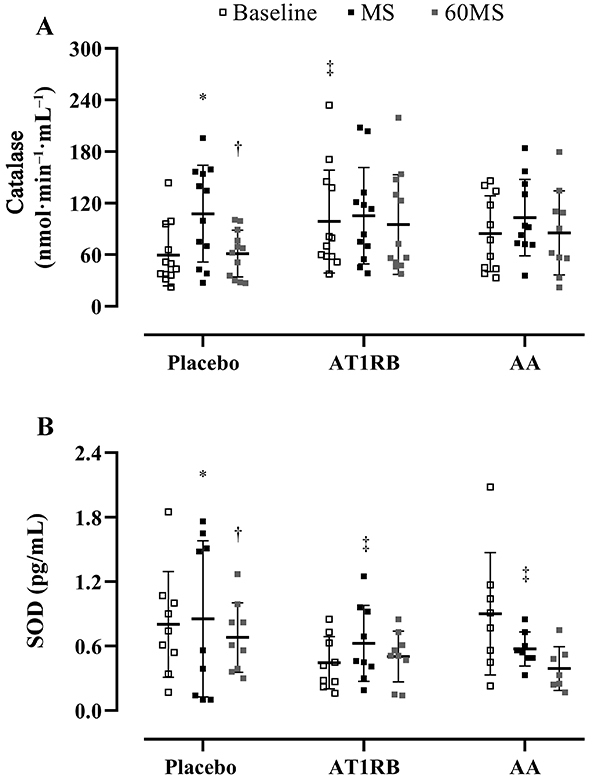
Catalase (**A**) and SOD (**B**) activity before (baseline), during (MS), and 60 min (60MS) after mental stress in overweight/obesity individuals after oral administration of placebo, AT1R blockade, and AA. Vertical lines indicate means and SD. *P<0.05 *vs* baseline; ^†^P<0.05 *vs* MS; ^‡^P<0.05 *vs* placebo at the same moment (ANOVA). FMD: flow-mediated dilation; AT1RB: angiotensin II type 1 receptor blockade; AA: ascorbic acid.

As for catalase activity, there was a baseline difference between placebo and AT1R blockade (placebo, 61.18±34.7 (nmol·min^-1^·mL^-1^) *vs* AT1R blockade, 120.27±96.2 pg/mL; P<0.05). During the placebo session, catalase activity increased during MS (baseline, 61.18±34.7 pg/mL *vs* MS, 116.24±61.9 pg/mL; P<0.01) and decreased to baseline levels 60 min after (MS, 116.24±61.9 pg/mL *vs* 60MS, 76.43±60.16 pg/mL; P<0.02). Once again, no differences were observed with either the AT1R blockade or with AA (Figure 4A). Comparable to catalase, SOD activity increased during MS only in the placebo session (baseline, 0.80±0.49 pg/mL *vs* MS, 0.85±0.72 pg/mL, P<0.01; placebo, 0.85±0.72 pg/mL *vs* AT1R blockade, 0.67±0.31 pg/mL, P<0.05; placebo *vs* AA, 0.48±0.22 pg/mL, P<0.05) and decreased to baseline levels 60 min after (MS, 0.85±0.72 pg/mL *vs* 60MS, 0.39±0.22 pg/mL; P<0.02) (Figure 4B).

## Discussion

The findings of the present study are three-fold: 1) AT1R blockade improved endothelial function after stressful situations in normotensive overweight/obese grade I men, reinforcing our hypothesis that the activation of the Ang II-AT1R pathway may be an important mechanism responsible for transient endothelial dysfunction; 2) ascorbic acid also improved endothelial function albeit only 1 h after exposure to stress; 3) stress provoked increases in both the oxidative profile (lipid peroxidation and protein carbonylation) and the antioxidant enzymes (catalase and SOD), while AT1R blockade and ascorbic acid prevented this response. Thus, the present study provided evidence that AT1R-mediated oxidative stress is an important underlying mechanism of transitory endothelial dysfunction induced by MS in overweight/obese adults.

Tasks of mental stress have been largely used as a simulation of mental or psychological stress situations in a standardized and controlled environment under hemodynamic, vascular, and electrocardiographic monitoring. Several studies, including from our research group, have used this type of intervention to assess endothelial function in healthy subjects and patients under cardiometabolic risk (3,5-7). The Stroop task used in the present study was able to inflict the same stressful stimulus in the three sessions, as evidenced by similar increases in hemodynamic variables during the protocol. However, to the best of our knowledge, ours is the first study to provide direct evidence of the participation of the Ang II-AT1R pathway in the impairment of endothelial function in response to MS.

In the present study, endothelial function was severely impaired 30 min after MS but recovered within 60 min in the placebo session. It is well documented that acute exposure to MS leads to transient endothelial dysfunction in healthy and pathological conditions (5,6); however, the magnitude and extent of this response can be influenced by the duration of MS (5), subjects' responsiveness (27), and previous health conditions (28). A previous study from our group showed that individuals with metabolic syndrome had reduced FMD at 30 and 60 min after acute MS (6). Considering that the subjects in this research did not present any other comorbidity, the recovery process may have been more efficient. Moreover, the return of FMD to baseline levels was accompanied by a normalization of the oxidative stress levels. It is possible that the normalization of the redox homeostasis is the mechanism behind the improvement in endothelial function.

On the other hand, AT1R blockade improved endothelial function after MS and this effect was maintained for up to 60 min, providing evidence that the Ang II-AT1R pathway is implicated in the transient endothelial function observed after MS. Indeed, the activation of the Ang II-AT1R pathway enhances the expression of ROCK1 and gp91^*phox*^, the catalytic component of NAPDH oxidase (29), which promotes oxidative stress and imbalances among vasoactive substances.

Ascorbic acid has been related to endothelial cell proliferation, apoptosis and smooth muscle-mediated vasodilation, among other endothelium-mediated effects (30). Clinical studies have shown that intravenous infusion of ascorbate promotes endothelial-dependent dilation in patients with cardiovascular risk such as atherosclerosis (31) and diabetes (32), possibly by sparing endothelial cell-derived NO and scavenging superoxide that would otherwise react with free NO (33). Corroborated by Plotnick et al. (34), AA did not influence endothelial function after mental stress, however, the maintenance of FMD at values similar to pre-stress could be interpreted as a protection/prevention mechanism against the transitory endothelial dysfunction observed during the placebo session. The improvement observed 60 min after MS could be a delayed effect of AA. It is important to highlight that Halliwill et al. (35) has shown that inhibition of the sympathetic system does not improve endothelial function during or after MS, reinforcing that oxidative stress may have a key role in modulating the endothelial response to MS.

Endothelial cells generate superoxide and hydrogen peroxide (H_2_O_2_) as a result of both cytoplasmic and mitochondrial metabolism (33). Moreover, NADPH oxidase activation, mainly by the stimulation of its subunits NOX1 and NOX4 (29), boosts the formation and accumulation of intracellular superoxide anion (36). The superoxide anion is rapidly dismutated to hydrogen peroxide, provoking endothelial cell damage (29). While ROS signaling is a key player in the maintenance of vascular tone, exposure to stressful situations seems to evoke imbalanced redox homeostasis, as observed in the present study. The MS task increased lipid peroxidation and protein carbonylation and increased catalase and SOD activity, possibly in response to a pro-oxidant environment. This phenomenon was neither observed when the Ang II- AT1R pathway was blocked nor when AA was infused. These findings supported our hypothesis that the transitory endothelial dysfunction observed after MS could be a result of A1TR-mediated redox imbalance.

Some limitations must be considered when interpreting the results of the present study. The lack of women in our sample may be considered a limitation concerning the external validity of the results to the entire population. In order to avoid the established effects of sex hormones on the vascular function of women, we opted to enroll only men in the study. Therefore, the present results do not allow us to infer that the same responses would be observed in women. Also, a group with eutrophic subjects would enrich the study, so our results cannot be extrapolated to this specific population either. Additionally, the lack of plasma Ang II measurements is a limitation. Regardless of the Ang II levels, the AngII-AT1R pathway may have a role in stress-mediated endothelial dysfunction even in normotensive overweight/obese adults. However, the 3-way crossover, randomized, placebo-controlled protocol may attenuate this limitation. During the experimental sessions, blind evaluators assessed all measures; however, a single non-blind evaluator analyzed all FMD data. Moreover, endothelium-independent vasodilation and AT1R blockade could not be tested; however, Stangier et al. (37) showed that 40 mg of telmisartan produced 80% inhibition of the receptor. Given that olmesartan presents higher binding affinity to the AT1R than telmisartan, it is unlikely that the inhibition induced in the present study was lower than that observed with telmisartan (38,39). Lastly, our results did not reflect changes *in vitro* and other studies are necessary for a better understanding of the mechanisms involved in this phenomenon.

In conclusion, the results of the present study provided compelling evidence regarding the transient endothelial dysfunction observed in response to acute MS. Moreover, the mentioned impairment in FMD seems to be directly influenced by the redox homeostasis imbalance. Blockade of the Ang II-AT1R pathway evoked a significant improvement in endothelial function after MS, while AA presented a delayed positive impact on flow-mediated dilation. Both interventions also prevented the deregulation of ROS signaling, providing evidence that A1TR-mediated oxidative stress is an important mediator of the FMD response after MS. Our study provides new insight into the mechanisms that underlie the deleterious response to MS and the MS consequence on the endothelial function of individuals with overweight/obesity. Thereby, defining new pathways that influence endothelial function is an important tool for understanding the pathological response to stressful situations.

## References

[1] Trachta P, Drapalova J, Kavalkova P, Touskova V, Cinkajzlova A, Lacinova Z (2014). Three months of regular aerobic exercise in patients with obesity improve systemic subclinical inflammation without major influence on blood pressure and endocrine production of subcutaneous fat. Physiol Res.

[2] Gonzalez-Muniesa P, Martinez-Gonzalez MA, Hu FB, Despres JP, Matsuzawa Y, Loos RJF (2017). Obesity. Nat Rev Dis Primers.

[3] Broadley AJM, Korszun A, Abdelaal E, Moskvina V, Jones CJH, Nash GB (2005). Inhibition of cortisol production with metyrapone prevents mental stress-induced endothelial dysfunction and baroreflex impairment. J Am Coll Cardiol.

[4] Musey PI, Schultebraucks K, Chang BP (2020). Stressing out about the heart: a narrative review of the role of psychological stress in acute cardiovascular events. Acad Emerg Med.

[5] Ghiadoni L, Donald AE, Cropley M, Mullen MJ, Oakley G, Taylor M (2000). Mental stress induces transient endothelial dysfunction in humans. Circulation.

[B06] Sales ARK, Fernandes IA, Rocha NG, Costa LS, Rocha HNM, Mattos JDM (2014). Aerobic exercise acutely prevents the endothelial dysfunction induced by mental stress among subjects with metabolic syndrome: the role of shear rate. Am J Physiol Heart Circ Physiol.

[7] Rocha NG, Sales AR, Miranda RL, Silva MS, Silva JF, Silva BM (2015). Aerobic exercise modulation of mental stress-induced responses in cultured endothelial progenitor cells from healthy and metabolic syndrome subjects. Life Sci.

[8] Geiker NRW, Astrup A, Hjorth MF, Sjödin A, Pijls L, Markus CR (2018). Does stress influence sleep patterns, food intake, weight gain, abdominal obesity and weight loss interventions and vice versa?. Obes Rev.

[9] Favero G, Paganelli C, Buffoli B, Rodella LF, Rezzani R (2014). Endothelium and its alterations in cardiovascular diseases: life style intervention. Biomed Res Int.

[10] Mehta PK, Griendling KK (2007). Angiotensin II cell signaling: physiological and pathological effects in the cardiovascular system. Am J Physiol Cell Physiol.

[11] Liu H, Kitazato KT, Uno M, Yagi K, Kanematsu Y, Tamura T (2008). Protective mechanisms of the angiotensin II type 1 receptor blocker candesartan against cerebral ischemia: *in-vivo* and *in-vitro* studies. J Hypertens.

[12] Widlansky ME, Gokce N, Keaney JF, Vita JA (2003). The clinical implications of endothelial dysfunction. J Am Coll Cardiol.

[13] Apovian CM, Gokce N (2012). Obesity and cardiovascular disease. Circulation.

[B14] Barton M, Carmona R, Ortmann J, Krieger JE, Traupe T (2003). Obesity-associated activation of angiotensin and endothelin in the cardiovascular system. Int J Biochem Cell Biol.

[B15] Celermajer DS, Sorensen KE, Gooch VM, Spiegelhalter DJ, Miller OI, Sullivan ID (1992). Non-invasive detection of endothelial dysfunction in children and adults at risk of atherosclerosis. Lancet.

[16] Tsai TH, Chai HT, Sun CK, Yen CH, Leu S, Chen YL (2012). Obesity suppresses circulating level and function of endothelial progenitor cells and heart function. J Transl Med.

[17] Pi-Sunyer X (2009). The medical risks of obesity. Postgrad Med.

[18] Simon GE, Arterburn D, Rohde P, Ludman EJ, Linde JA, Operskalski BH (2011). Obesity, depression, and health services costs among middle-aged women. J Gen Intern Med.

[19] Ministério da Saúde DATASUS. Mortalidade por doenças do aparelho circulatório (CID-10) 2011. http://tabnet.datasus.gov.br/cgi/tabcgi.exe?sim/cnv/obt10uf.def.

[20] Schwocho LR, Masonson HN (2001). Pharmacokinetics of CS-866, a new angiotensin II receptor blocker, in healthy subjects. J Clin Pharmacol.

[21] Thijssen DH, Black MA, Pyke KE, Padilla J, Atkinson G, Harris RA (2011). Assessment of flow-mediated dilation in humans: a methodological and physiological guideline. Am J Physiol Heart Circ Physiol.

[22] Stroop JR (1935). Studies of interference in serial verbal reactions. J Experimental Psychol.

[23] Fernandes IA, Sales ARK, Rocha NG, Silva BM, Vianna LC, da Nobrega ACL (2014). Preserved flow-mediated dilation but delayed time-to-peak diameter in individuals with metabolic syndrome. Clin Physiol Funct Imaging.

[24] Tsikas D (2017). Assessment of lipid peroxidation by measuring malondialdehyde (MDA) and relatives in biological samples: analytical and biological challenges. Anal Biochem.

[25] Lowry OH, Rosebrough NJ, Farr AL, Randall RJ (1951). Protein measurement with the Folin phenol reagent. J Biol Chem.

[26] Atkinson G, Batterham AM (2013). Allometric scaling of diameter change in the original flow-mediated dilation protocol. Atherosclerosis.

[27] Carter JR, Ray CA (2009). Sympathetic neural responses to mental stress: responders, nonresponders and sex differences. Am J Physiol Heart Circ Physiol.

[28] Loures DL, Sant Anna I, Baldotto CSR, de Sousa EB, da Nobrega ACL (2002). Mental stress and cardiovascular system [in Portuguese]. Arq Bras Cardiol.

[29] Chung IM, Kim YM, Yoo MH, Shin MK, Kim CK, Suh SH (2010). Immobilization stress induces endothelial dysfunction by oxidative stress via the activation of the angiotensin II/its type I receptor pathway. Atherosclerosis.

[30] May JM, Harrison FE (2013). Role of vitamin C in the function of the vascular endothelium. Antioxid Redox Signal.

[31] Tousoulis D, Xenakis C, Tentolouris C, Davies G, Antoniades C, Crake T (2005). Effects of vitamin C on intracoronary L-arginine dependent coronary vasodilatation in patients with stable angina. Heart.

[32] Tousoulis D, Antoniades C, Vasiliadou C, Kourtellaris P, Koniari K, Marinou K (2007). Effects of atorvastatin and vitamin C on forearm hyperaemic blood flow, asymmentrical dimethylarginine levels and the inflammatory process in patients with type 2 diabetes mellitus. Heart.

[33] May JM (2000). How does ascorbic acid prevent endothelial dysfunction?. Free Radic Biol Med.

[34] Plotnick MD, D'Urzo KA, Gurd BJ, Pyke KE (2017). The influence of vitamin C on the interaction between acute mental stress and endothelial function. Eur J Appl Physiol.

[35] Halliwill JR, Lawler LA, Eickhoff TJ, Dietz NM, Nauss LA, Joyner MJ (1997). Forearm sympathetic withdrawal and vasodilatation during mental stress in humans. J Physiol.

[36] Nauseef WM (2008). Biological roles for the NOX family NADPH oxidases. J Biol Chem.

[37] Stangier J, Su CA, van Heiningen PN, Meinicke T, van Lier JJ, de Bruin H (2001). Inhibitory effect of telmisartan on the blood pressure response to angiotensin II challenge. J Cardiovasc Pharmacol.

[38] Meredith P (2010). Comparative ARB pharmacology. Br J Cardiol.

[39] Miura S, Karnik SS, Saku K (2011). Review: angiotensin II type 1 receptor blockers: class effects *versus* molecular effects. J Renin Angiotensin Aldosterone Syst.

